# Mass Testing and Treatment to Accelerate Malaria Elimination: A Systematic Review and Meta-Analysis

**DOI:** 10.4269/ajtmh.23-0127

**Published:** 2024-03-12

**Authors:** Beena Bhamani, Elisabet Martí Coma-Cros, Maria Tusell, Vita Mithi, Elisa Serra-Casas, Nana Aba Williams, Kim A. Lindblade, Koya C. Allen

**Affiliations:** ^1^ Barcelona Institute for Global Health (ISGlobal), Hospital Clínic – Universitat de Barcelona, Barcelona, Spain;; ^2^ Armref Data for Action in Public Health Research Consultancy, Mzuzu, Malawi;; ^3^ Society for Research on Nicotine and Tobacco-Genetics and Omics Network, Madison, Wisconsin;; ^4^ Leaders of Africa Institute, Baltimore, Maryland;; ^5^ Global Malaria Programme, World Health Organization, Geneva, Switzerland

## Abstract

In regions where malaria transmission persists, the implementation of approaches aimed at eliminating parasites from the population can effectively decrease both burden of disease and transmission of infection. Thus, mass strategies that target symptomatic and asymptomatic infections at the same time may help countries to reduce transmission. This systematic review assessed the potential benefits and harms of mass testing and treatment (MTaT) to reduce malaria transmission. Searches were conducted in March 2021 and updated in April 2022 and included cluster-randomized controlled trials (cRCTs) as well as nonrandomized studies (NRSs) using malaria infection incidence, clinical malaria incidence, or prevalence as outcomes. The risk of bias was assessed with Cochrane’s risk of bias (RoB2) tool and Risk of Bias Tool in Nonrandomized Studies – of Interventions (ROBINS-I), and the certainty of evidence (CoE) was graded for each outcome. Of 4,462 citations identified, seven studies (four cRCTs and three NRSs) contributed outcome data. The analysis revealed that MTaT did not reduce the incidence (risk ratio [RR]: 0.95, 95% CI: 0.87–1.04; 1,181 participants; moderate CoE) or prevalence (RR: 0.83, 95% CI: 0.67–1.01; 7,522 participants; moderate CoE) of malaria infection but resulted in a small reduction in clinical malaria (RR: 0.82; 95% CI: 0.70–0.95; 334,944 participants; moderate CoE). Three studies contributing data on contextual factors concluded that MTaT is an acceptable, feasible, and cost-effective intervention. Mathematical modeling analyses (*n =* 10) suggested that MTaT effectiveness depends on the baseline transmission level, diagnostic test performance, number of rounds, and other co-interventions. Based on the limited evidence available, MTaT has little to no impact on reducing malaria transmission.

## INTRODUCTION

With progress in malaria control stalled and funding levels essentially unchanged since 2010,^1^ the global malaria community is seeking cost-effective interventions to reduce malaria transmission and get back on track to achieve global targets. Reducing the reservoir of the malaria parasite in the human host necessitates identifying and eliminating all forms of the parasite carried by both symptomatic and asymptomatic individuals. To eliminate malaria, initiatives should focus on identifying and eliminating infection clusters through a combination of passive and active case detection methods. Passive case detection (PCD) involves identifying malaria cases among individuals who voluntarily seek treatment,^2^^,^^3^ whereas active case detection (ACD) refers to the identification of malaria cases in the community and households by health workers, often targeting specific population groups considered to be at high risk.^2^^,^^3^ Active case detection strategies that identify “silent” infections in persons who are unlikely to seek diagnosis and treatment have become more popular as a result of the increasing recognition of asymptomatic infections as important reservoirs of infection.^4^ Numerous studies have demonstrated that undetectable gametocytemia as low as 5 gametocytes (g)/μL can infect mosquitoes and contribute to malaria transmission.^5^ Malaria infections, even those that are asymptomatic, have a role in maintaining malaria transmission and also have significant deleterious effects on health and society. Hence, population-wide strategies such as mass testing and treatment (MTaT), which involves testing the entire population living in a delimited geographic area and treating all positive cases,^2^ and mass drug administration (MDA), which involves the administration of antimalarial medicines to an entire population located in a defined geographic area regardless of infection status,^2^ need additional assessment and evaluation to determine their effectiveness on impact in undetectable malaria cases.^4^^,^^5^

Mass drug administration involves administering antimalarials without testing, and concerns have been raised around the safety of this intervention, including the potential risk of developing drug resistance.^6^ An alternative approach to achieving malaria elimination in areas with ongoing transmission is the implementation of an MTaT strategy. This approach is centered around actively identifying infections, whether they are symptomatic or asymptomatic, and offers potential advantages over MDA by reducing the unnecessary use of antimalarial medications in individuals who do not require treatment. This may, in turn, lower the risk of antimalarial drug resistance.^5^^,^^7^ Furthermore, antimalarial drugs, including artemisinin-based combination therapy, are often expensive and may be available in limited quantities. Consequently, expending these resources on individuals without malaria can incur significant costs,^8^ thus suggesting that MTaT may be a more effective, acceptable, and feasible alternative intervention.

In 2015, the WHO convened an evidence review group to examine the evidence for MDA, MTaT, and focal screening and treatment (FSAT) for malaria. The objective was to examine evidence on whether these strategies could interrupt malaria transmission. On the basis of this review, the WHO Malaria Policy Advisory Committee concluded that there was a lack of evidence recommending MTaT and FSAT for interrupting malaria transmission, whereas MDA was recommended only in areas approaching elimination and during an epidemic or complex emergencies.^7^

The purpose of this systematic review was to assess the current evidence for impacts of MTaT on malaria transmission to support the WHO guideline development process. We sought to 1) determine the benefits and harms of parasitological testing of adults and children residing in a delimited geographic area (with ongoing transmission or malariogenic potential) at the same time and treating all confirmed cases with a full therapeutic course of an antimalarial medicine, including radical treatment of *Plasmodium vivax*, compared with no intervention; 2) identify contextual factors that might favor or disadvantage MTaT as a public health intervention, including values and preferences of end users, health equity, acceptability, feasibility, and resource requirements; 3) assess the factors that may epidemiologically modify the effects of MTaT (known as potential effect modifiers); and 4) assess and summarize insights from mathematical modeling studies. After this review, the WHO Guideline Development Group (GDG) reconvened in 2021 and subsequently revised its guidelines in 2022.^9^^,^^10^

## MATERIALS AND METHODS

This systematic review and meta-analysis adhered to the Preferred Reporting Items for Systematic Reviews and Meta-Analyses (PRISMA) guidelines,^11^ and the protocol was registered in the International Prospective Register of Systematic Reviews (PROSPERO; CRD42021259606).^12^ The full systematic review methodology has been described in detail elsewhere,^13^ and a brief summary of the methodology is given here.

### Population, intervention, comparison, and outcomes.

Mass testing and treatment was defined as parasitological testing of an entire population and treatment of confirmed cases with a full course of antimalarial medicine, including treatment of *P. vivax* and *Plasmodium ovale* liver-stage parasites at approximately the same time. The population was adults and children residing in a delimited geographic area with ongoing human malaria transmission or malariogenic potential. The comparator was no MTaT.

Key outcomes of interest for the review included incidence of malaria infection (measured using active surveillance) at the community level; prevalence of infection (point prevalence of malaria parasitemia) at the community level; incidence of clinical malaria (measured using passive surveillance/data from health care settings); elimination (defined as zero indigenous or local cases for a period during the transmission season); prevalence of drug resistance markers; incidence of adverse events (AEs) among the group targeted by the intervention; and prevalence of infection among the group targeted by the intervention as a proxy indicator of the potential effect at the community level. These outcomes are defined elsewhere in further detail.^13^

### Search strategy, selection, data extraction, and analysis.

The search strategy for this review is presented in Supplemental Table 1. Briefly, the first literature search was conducted in March 2021 and updated in April 2022. Title and abstract screenings were independently conducted by four reviewers in pairs (B. B., V. M., E. M. C., and K. C. A.), and the full texts of the potentially eligible articles were independently reviewed by three reviewers (B. B., V. M., and K. C. A.). Initially, it was planned that two authors would conduct full text screening, but an additional author was included owing to the large number of studies included in the title/abstract screening. The selection criteria, data extraction, assessment of heterogeneity, and statistical analysis followed during this systematic review are presented in detail elsewhere.^13^

Studies used different names and descriptions to refer to the MTaT intervention (i.e., mass screening and treatment, mass testing, treatment and tracking, mass surveillance and treatment, and community-wide screening and treatment); however, for clarity MTaT will be used throughout this paper. See Table 1 for names and abbreviations used by the different studies included in this review.

**Table 1:** Characteristics of included studies

Study	Location	Year(s)	Study Design	Intervention	Outcomes Reported
Samuels et al.^18^ Desai et al.^17^	Siaya, western Kenya	2013–2015	cRCT	Target population: all inhabitants Intervention: MTaT with RDTs Comparator: standard of care Drug: DP/AL/quinine Rounds: six (three per year over 2 years)	Incidence of infection at the community level (cohort followed monthly throughout the year) Prevalence of infection at the community level (2 months after intervention) Incidence of clinical malaria at the community level (follow-up range September 2013–September 2015)
Sutanto et al.^19^	Wesiku, West Timor, Indonesia	2013	cRCT	Target population: all inhabitants Intervention: MST with microscopy Comparator: no MST/standard of care Drug: DP/primaquine Rounds: MST 3: three (every 5 weeks) MST 2: two (every 10 weeks)	Incidence of infection at the community level (children followed up monthly for 6 months) AEs (followed daily for 6 months)
Larsen et al.^20^	Southern Zambia	2012–2013	cRCT	Target population: all inhabitants Intervention: MTaT with RDTs Comparator: standard of care Drug: AL Rounds: three (every 2 months)	Incidence of clinical malaria at the community level (health facility data reported monthly throughout the year) Prevalence of infection at the community level (6 months after intervention)
Tiono et al.^21^	Saponé, Burkina Faso	2011–2012	cRCT	Target population: all inhabitants Intervention: mass screening and treatment with RDTs Comparator: standard of care Drug: AL Rounds: three (1 month apart)	Incidence of clinical malaria at the community level (children followed up for 1 year from round 1) AEs (reported within 7 days) SAEs (within 30 days of any treatment) Prevalence of infection at the community level (9 months after intervention)
Linn et al.^22^	Senegal	2013	NRS	Target population: every household Intervention: ProACT sweeps with RDTs Comparator: standard of care Drug: ACT (per national policy) Rounds: 21 (weekly)	Prevalence of infection at the community level (symptomatic infection–week 21 of intervention)
Ndong et al.^23^	Pakro, Ghana	2017–2018	uBAF	Target population: all inhabitants Intervention: MTTT with RDTs Comparator: N/A Drug: AA/AL Rounds: four rounds (every fourth month)	Prevalence of infection at the community level (asymptomatic parasitemia–4 months after intervention) Prevalence of infection at the community level (symptomatic parasitemia–4 months after intervention)
Bharti et al.^24^	Mandla, India	2017–2020	uBAF	Targeted population: all inhabitants Intervention: ITN, IRS, active case management, and MSAT with RDTs Comparator: N/A Drug: ACT (per national drug policies) Rounds: three rounds not in the same population (in different API areas)	Prevalence of infection at the community level (in different API areas to assess active and passive surveillance systems)

AA = artesunate-amodiaquine; ACT = artemisinin-based combination therapy; AE = adverse event; AL = artemether-lumefantrine; API = annual parasite incidence; cRCT = cluster-randomized controlled trial; DP = dihydroartemisinin-piperaquine; IRS = indoor residual spraying; ITN = insecticide-treated net; MSAT = mass surveillance and treatment; MST = mass screening and treatment; MTaT = mass testing and treatment; MTTT = mass testing, treatment, and tracking; N/A = not applicable; NRS = nonrandomized study; ProACT = proactive community treatment; RDT = rapid diagnostic test; SAE = serious adverse event; uBAF = uncontrolled before and after.

Detailed methods for determining the risk of bias are described elsewhere.^13^ Briefly, for cluster-randomized controlled trials (cRCTs), the Cochrane risk of bias (RoB2) tool was used as described in Higgins et al.,^14^ and for nonrandomized studies (NRSs), the Risk of Bias Tool in Nonrandomized Studies – of Interventions (ROBINS-I) tool was used as described in Sterne et al.^15^ Uncontrolled before-and-after studies and before-and-after studies with historical controls were judged to have a critical risk of bias. The Grading of Recommendations, Assessment, Development and Evaluation method was used to assess the certainty of evidence across each outcome measure.^16^

## RESULTS

### Study selection.

A total of 4,462 records were identified: 4,374 via searching databases, 48 from registers, and 40 via other methods (e.g., websites, organizations, and citation searching). Before the screening, 1,480 records were removed because of duplication, with a total of 2,982 records to be screened against title and abstract for eligibility (2,942 identified via databases and registers, and 40 identified via other methods). Of these, 101 were assessed using the full text. Seventy-seven articles were excluded after full-text screening for the following reasons: incorrect intervention (*n =* 8), incorrect population (*n =* 1), incorrect study focus area (*n =* 3), incorrect study design (*n =* 6), cross-referenced article (*n =* 7), no reported outcomes of interest (*n =* 1), reports excluded for contextual factors (*n =* 6), and reports excluded for mathematical modeling (*n =* 4) or because a full report could not be retrieved (e.g., abstract, protocol, or other document and ongoing studies) (*n =* 41). All full-text studies that did not meet eligibility criteria are listed in Supplemental Table 2 with the reasons for exclusion.

**Figure 1.**
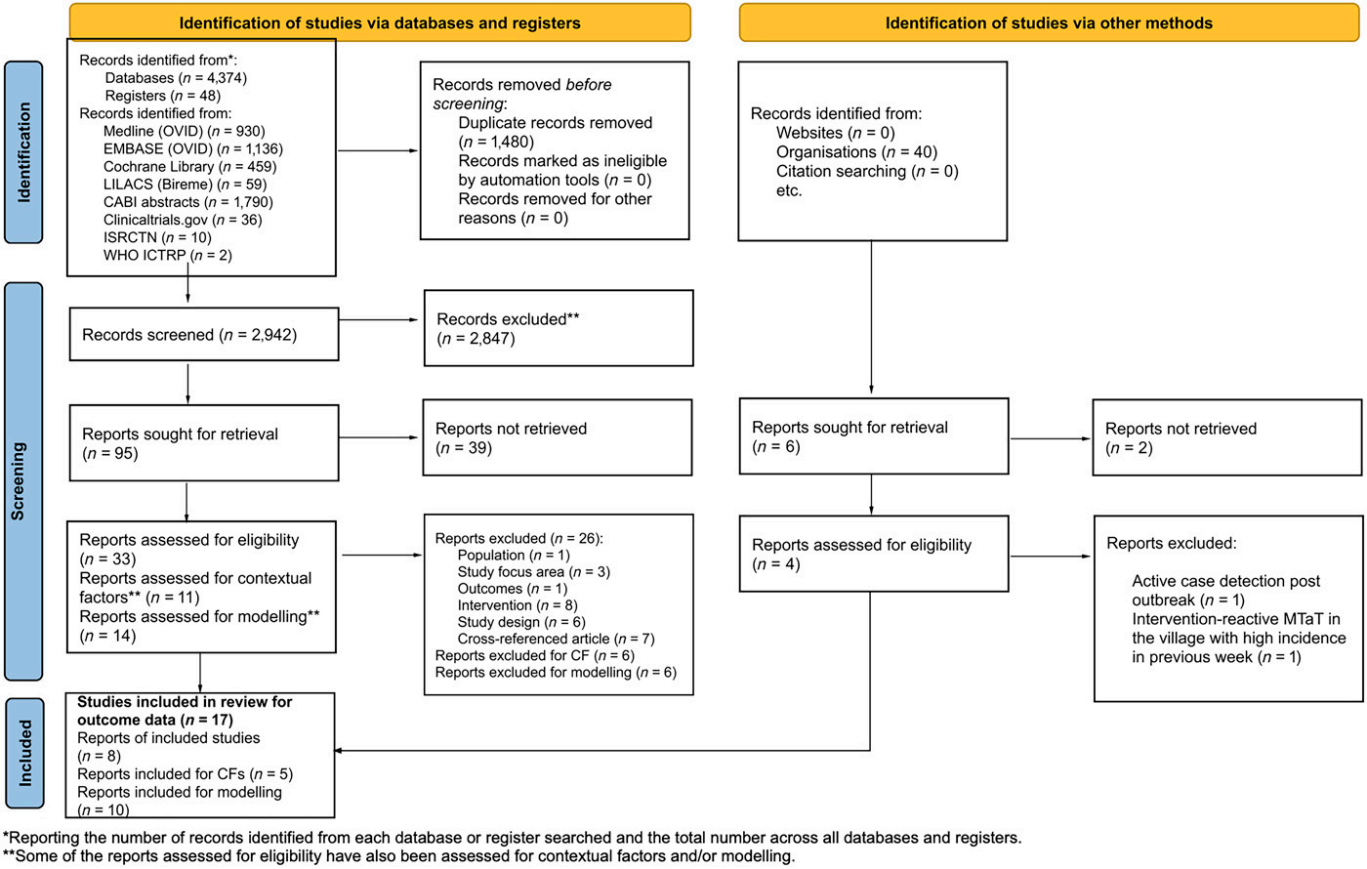
PRISMA flow diagram for systematic review of mass test and treat (MTaT) for reduction of human malaria. CF = contextual factors; ICTRP = International Clinical Trials Registry Platform; ISRCTN = International Standard Randomised Controlled Trial Number; PRISMA = Preferred Reporting Items for Systematic Reviews and Meta-Analyses.

After the full-text screening, seven studies were included for assessment of outcome data (eight reports), three studies were included for assessment of contextual factors (five reports), and 10 studies were included for mathematical modeling. The detailed PRISMA Flow Diagram is presented in Figure 1, which also includes the reasons for exclusion. Of the seven studies that met the criteria for inclusion, four were cRCTs conducted in Kenya,^17^^,^^18^ Indonesia,^19^ Zambia,^20^ and Burkina Faso^21^, and three were NRSs, including one nonrandomized, cluster-controlled trial conducted in Senegal^22^ and two uncontrolled before-and-after studies conducted in Ghana^23^ and India.^24^ Descriptive characteristics of the seven included studies are summarized in Table 1 and described in detail in Supplemental Table 3. Among the five articles providing information on contextual factors, two articles assessed acceptability,^25^^,^^26^ one article evaluated resource use,^27^ and two articles assessed the feasibility of MTaT.^28^^,^^29^ The 10 mathematical modeling studies included in this review were categorized as simulations.^8^^,^^30^^–^^38^

All seven studies^17^^–^^24^ with outcome data reported targeting the entire population residing in a delimited geographical area (i.e., the villages, hamlets, or subdistricts selected for the intervention). Studies varied in transmission settings: three of the four cRCTs^17^^,^^18^^,^^20^^,^^21^ were conducted in moderate- to high-transmission settings, whereas the fourth cRCT^19^ was conducted in a low-transmission area. The NRS in Senegal was implemented in an area of moderate transmission,^22^ whereas the two uncontrolled before-and-after studies were conducted in a high-transmission setting of Ghana^23^ and in an area of low transmission in India.^24^

The cRCTs implemented two to three rounds of MTaT per year^17^^–^^21^; three of the studies implemented 1 year of MTaT, whereas the study in western Kenya conducted MTaT over 2 years. The NRS in Senegal implemented 21 weekly rounds of MTaT over the transmission season.^22^ In the two uncontrolled before-and-after studies, one^23^ assessed the impact of four MTaT rounds implemented every 4 months, whereas the other study^24^ conducted just one round of MTaT across three different transmission settings. Almost all studies^17^^,^^18^^,^^20^^–^^24^ deployed rapid diagnostic tests (RDTs) to provide parasitological confirmation, except for one study^19^ that used microscopy. The antimalarials used varied across the studies and followed each country’s national malaria guidelines.

### Risk of bias in studies.

Supplemental Figures 1 and 2 summarize risk of bias assessments for the outcomes in the four cRCTs.^17^^–^^21^ All outcomes of interest in the studies were assessed and rated as low risk of bias for all domains except for the selection of reported data. For AEs and serious AEs (SAEs), two studies^19^^,^^21^ were rated as having some concerns due to reporting of cumulative outcomes not separated by study intervention and control arms.

The risk of bias assessment for the quasi-experimental study^22^ is summarized in Supplemental Figure 3. Bias due to confounding was rated as serious because the potential confounding due to the gold rush was not accounted for in the analysis because of the unavailability of data. Bias due to missing data was rated as moderate because of missing registers from health facilities in the intervention village, and the analysis is unlikely to have minimized the risk of bias arising from the missing data. All other domains were rated as low risk of bias.

No risk of bias tool was used for the two uncontrolled before-and-after studies.^23^^,^^24^ Owing to the inherent biases associated with the study design, both studies were rated as critical overall risk of bias.

### Results by outcome.

**Figure 2.**
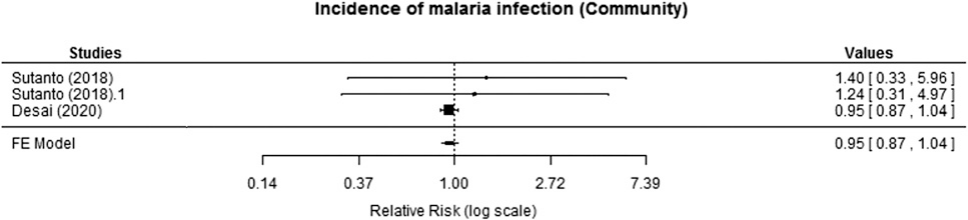
Effect of mass testing and treatment versus no mass testing and treatment on incidence of malaria infection at the community level (cluster-randomized controlled trials [cRCTs]). FE = fixed effects.

Two cRCTs reported incidence of malaria infection at the community level. Desai et al.^17^ followed a cohort monthly for 1 year using active surveillance and found similar annual cumulative incidence of all malaria infections between the intervention and control arms (crude incidence rate ratio [IRR]: 0.95, 95% CI: 0.87–1.04). Sutanto et al.^19^ followed a cohort of children for 6–12 months and assessed the incidence of malaria infection in the two intervention arms (MTaT with two rounds [MTaT2] and MTaT with three rounds [MTaT3]) compared with the control arm (MTAT0) for 6 months. The risk ratio (RR) reported for MTaT3 or MTaT2 compared with the control arm showed no significant difference in malaria infection (MTaT3 versus MTaT0: RR: 1.24, 95% CI: 0.31–4.97; MTaT2 versus MTaT0: RR: 1.40, 95% CI: 0.33–5.96). The meta-analysis also revealed that the intervention likely results in little to no difference in the incidence of malaria infection at the community level (RR: 0.95, 95% CI: 0.87–1.04; moderate certainty of evidence; Figure 2).

**Figure 3.**
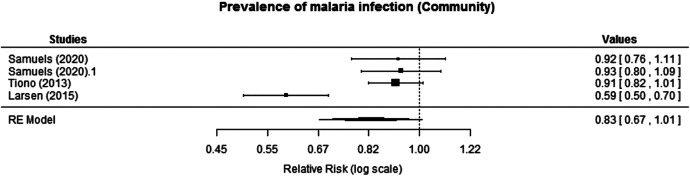
Effect of mass testing and treatment versus no mass testing and treatment on prevalence of infection at the community level (cluster-randomized controlled trials [cRCTs]). RE = random effects.

Three cRCTs reported prevalence of malaria at the community level. Samuels et al.^18^ reported prevalence of malaria infection at the community level at 2 months after intervention for year 1 and for year 2 after three annual MTaT rounds. The effect size of the intervention on prevalence was not significant after year 1 (adjusted ratio of prevalence ratio [aRPR]: 0.93, 95% CI: 0.80–1.09) or year 2 (aRPR: 0.92, 95% CI: 0.76–1.11). Tiono et al.^21^ reported prevalence of infection in asymptomatic carriers at 9 months after intervention, and there was no statistically significant difference between the intervention and control arms (RR: 0.91, 95% CI: 0.82–1.01). Larsen et al.^20^ reported prevalence of infection in children (1–59 months) at 6 months after intervention. The prevalence reported was lower in the intervention areas, suggesting that MTaT significantly decreased the odds of malaria in the targeted population compared with the control (adjusted odds ratio [aOR]: 0.47, 95% CI: 0.24–0.90). A fixed-effects model was used for the meta-analysis; however, the I^2 value (86.4%) indicated a high degree of heterogeneity between the studies reporting prevalence outcomes. Although Larsen et al.^20^ demonstrated a statistically significant reduction, the remaining three studies^18^^,^^21^ exhibited comparable point estimates and overlapping CIs, showing no impact of MTaT on malaria prevalence. Conducting a subgroup analysis to explore potential sources of heterogeneity was not feasible because of the limited number of studies. Given the pronounced heterogeneity, a random effects model was considered appropriate. The meta-analysis showed no significant reduction in malaria prevalence (RR: 0.83, 95% CI: 0.67–1.01; moderate certainty of evidence; Figure 3). However, given the considerable heterogeneity between the studies, it is advisable to approach the interpretation of the findings with caution.

**Figure 4.**
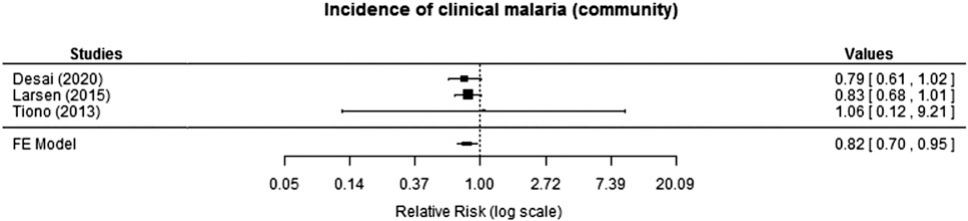
Effect of mass testing and treatment versus no mass testing and treatment on incidence of clinical malaria at the community level (cluster-randomized controlled trials [cRCTs]). FE = fixed effects.

Three cRCTs reported incidence of clinical malaria at the community level. Desai et al.^17^ reported a 21% reduction in incidence over the 2-year study period, which was assessed using passive surveillance, but the reduction was not statistically significant (IRR: 0.79, 95% CI: 0.61–1.02). Similarly, a marginal reduction in incidence of clinical malaria during the intervention period (12 months) was reported by Larsen et al.^20^ (IRR: 0.83, 95% CI: 0.68–1.01). Likewise, the incidence of clinical malaria reported in Tiono et al.^21^ did not exhibit a statistically significant difference between the intervention and control arms; mean number of confirmed malaria cases per person-year was 1.69 versus 1.60 (*P* = 0.3482; RR: 1.06; 95% CI: 0.12–9.21). When the effects were combined for meta-analysis, the pooled effect showed some protective effect of the intervention, with an 18% reduction in incidence of clinical malaria (RR: 0.82, 95% CI: 0.70–0.95; moderate certainty of evidence; Figure 4).

Two studies reported AEs and/or SAEs. Tiono et al.^21^ reported SAEs monitored from the time of consent until 30 days after the last dose of treatment and AEs during a period of 7 days after treatment administration. The study reported no significant differences in SAEs and AEs between the intervention and control arms. However, data in the report did not specify SAEs and AEs separately for intervention and control arms; thus, estimating the effect size was not plausible. Sutanto et al.^19^ reported no SAEs by the treatment assessed at the health centers, and no withdrawals of subjects were observed because of SAEs. The effect size was not presented here owing to cumulative data reporting in the study. The common AEs noted included fever (0.023/person-day), headache (0.008/person-day), vomiting (0.006/person-day), cough (0.004/person-day), shivering (0.003/person-day), and nasal congestion (0.002/person-day).

Prevalence of infection was also reported in one quasi-experimental and uncontrolled before-and-after study. The quasi-experimental study by Linn et al.^22^ screened and treated symptomatic cases to assess the impact on prevalence of symptomatic malaria after week 21 of the intervention and found lower odds of symptomatic malaria in those targeted by the intervention (aOR: 0.03, 95% CI: 0.02–0.07; very low certainty of evidence). Likewise, the uncontrolled before-and-after study conducted by Ndong et al.^23^ tested and treated asymptomatic cases only and reported prevalence of asymptomatic parasitemia at 4 months after the last round of the intervention. The symptomatic cases were also reported and treated as part of the standard of care at public health centers in the community. It was found that after four rounds of MTaT, the odds of asymptomatic parasitemia was reduced by 24% (aOR: 0.76, 95% CI: 0.67–0.85; very low certainty of evidence), whereas the odds of symptomatic parasitemia was reduced by 9% over that same period and was not statistically significant (aOR: 0.91, 95% CI: 0.67–1.38; very low certainty of evidence). The second uncontrolled before-and-after study conducted by Bharti et al.^24^ did not implement MTaT in the same population during the whole duration of the study; rather, each of the three rounds was administered in a different study area with a different transmission intensity, and prevalence was compared with the prevalence measured by passive surveillance in corresponding transmission settings. In the first round, conducted in a moderate– to high–annual parasite incidence (API) area, prevalence in the MTaT arm was 0.18% compared with 0.06% through passive surveillance during the same time period. The second round, conducted in low- and high-API areas showed 0.06% prevalence using MTaT compared with 0.03% prevalence in passive surveillance. In the last round, which was conducted in low-API areas around the cryptic cases, “where epidemiological investigations fail to identify an apparent mode of acquisition,”^39^ only 0.03% prevalence was measured through MTaT. The summary of findings on MTaT compared with no MTaT for reduction of malaria transmission is provided in Table 2.

**Table 2:** GRADE Summary of findings

Outcomes	Anticipated Absolute Effects* (95% CI)		Relative Effect (95% CI)	No. of Participants (studies)	Certainty of the Evidence (GRADE)	Comments
	Risk with No MTaT	Risk with MTaT				
0–12 months: incidence of malaria infection	1,646 per 1,000	1,564 per 1,000 (1,432–1,712)	Rate ratio: 0.95 (0.87–1.04)	1,296 (Two RCTs)^17^^,^^19^	⨁⨁⨁◯ Moderate^†^	MTaT likely results in little to no difference in incidence of malaria infection
2–9 months: prevalence of infection	379 per 1,000	329 per 1,000 (254–382)	RR: 0.83 (0.67–1.01)	7,309 (Three RCTs)^18^^,^^20^^,^^21^	⨁⨁⨁◯ Moderate^‡^	MTaT results in little to no difference in prevalence of malaria infection
0–12 months: incidence of clinical malaria	242 per 1,000	199 per 1,000 (170–230)	RR: 0.82 (0.70–0.95)	334,944 (Three RCTs)^17^^,^^20^^,^^21^	⨁⨁⨁◯ Moderate	MTaT likely reduces incidence of clinical malaria slightly
AE (group targeted by the intervention)	Most common AEs during treatment were fever (0.023/person-day), headache (0.008/person-day), vomiting (0.006/person-day), cough (0.004/person-day), shivering (0.003/person-day), and nasal congestion (0.002/person-day)			(One RCT)^19^	⨁⨁◯◯ Low^§^	The evidence is very uncertain about the effect of MTaT on AEs
SAE (group targeted by the intervention)	0 per 1,000	0 per 1,000 (0–0)	Not estimable	6,373 (One RCT)^21^	⨁⨁◯◯ Low^‖^^¶^	The evidence is very uncertain about the effect of MTaT on SAEs
Prevalence of infection (NRS)	Three rounds of MTaT were conducted to determine prevalence in the asymptomatic reservoir; MTaT was compared with detection through passive surveillance prevalence; first round: moderate- to high-burden areas−50/28,527 (i.e., 0.18% vs. 0.06% from passive surveillance); second round: low- to high-burden areas−7/11,363 (i.e., 0.06% vs. 0.03% from passive surveillance); third round: RCD of cryptic cases in 50 households −3/8,467 (i.e., 0.03%)			(One observational study)^24^	⨁◯◯◯ Very low^#^	The evidence is very uncertain about the effect of MTaT on the prevalence of infection
2 months: prevalence of infection (NRS)	34 per 1,000	1 per 1,000 (1–2)	OR: 0.03 (0.02–0.07)	8,508 (One observational study)^22^	⨁◯◯◯ Very low^#^**^†^^†^	The evidence is very uncertain about the effect of MTaT on the prevalence of infection
12 months: prevalence of infection (NRS)	438 per 1,000	415 per 1,000 (343–519)	OR: 0.91 (0.67–1.38)	416 (One observational study)^23^	⨁◯◯◯ Very low^‡^^#^	The evidence is very uncertain about the effect of MTaT on the prevalence of infection
12 months: prevalence of infection (NRS)	363 per 1,000	302 per 1,000 (277–327)	OR: 0.76 (0.67–0.85)	8,907 (One observational study)^23^	⨁◯◯◯ Very low^‡^^#^	The evidence is very uncertain about the effect of MTaT on the prevalence of infection

AE = adverse event; GRADE = Grading of Recommendations, Assessment, Development and Evaluation; MTaT = mass testing and treatment; NRS = nonrandomized study; OR = odds ratio; RCT = randomized controlled trial; RR = risk ratio; SAE = serious adverse event. To achieve transparency and implicity, the GRADE system classifies the certainty of evidence in one of four grades: High: Further research is very unlikely to change our confidence in the estimate of effect. ⨁⨁⨁⨁ Moderate: Further research is likely to have an important impact on our confidence in the estimate of effect and may change the estimate. ⨁⨁⨁◯ Low: Further research is very likely to have an important impact on our confidence in the estimate of effect and is likely to change the estimate. ⨁⨁◯◯ Very low: Any estimate of effect is very uncertain. ⨁◯◯◯

* The risk in the intervention group (and its 95% confidence interval) is based on the assumed risk in the comparison group and the relative effect of the intervention (and its 95% CI).

^†^ One study had two intervention arms. Both intervention arms were pooled with another study and compared with the control. The control arm was inflated in value because it was the same comparison group for the two different intervention arms in one study.

^‡^ Used as a proxy for prevalence of infection at the community level.

^§^ SAEs and AEs are not classified based on intervention and control arms.

^‖^ Unable to calculate control measures in the absence of control measure.

^¶^ Common AEs are reported for the whole study; however, no breakdown is provided for different arms.

^#^ Critical overall risk of bias due to inherent biases associated with study design.

** Fever and symptoms screening before testing.

^††^ Study did not control for one confounding domain and missing register from health facility in intervention village; the analysis is unlikely to have removed the risk of bias arising from the missing data.

^††^ Study did not control for one confounding domain and missing register from health facility in intervention village; the analysis is unlikely to have removed the risk of bias arising from the missing data.

### Contextual factors.

The acceptability of the intervention was reported in two qualitative studies: one by Shuford et al.,^26^ which was linked to a study conducted in western Kenya,^17^^,^^18^ and the other by Silumbe et al.,^25^ which was linked to the study conducted in southern Zambia^20^ and explored the perception of intermittent MTaT in the community. Both studies found similar responses in the post-implementation round, including fear of covert HIV testing and failed treatment adherence. Silumbe et al.^25^ also aimed to understand the perception of community health workers (CHWs) regarding the intervention. In general, MTaT was perceived very positively by most of the CHWs, except for some barriers including imperfect transportation to access hard-to-reach areas, difficulty in charging personal digital devices for data collection owing to unavailability of charging sources, and commodity shortages.

Data on resource use for MTaT was abstracted from one publication^27^ linked to the implementation of population-wide MTaT in southern Zambia.^20^ The authors concluded that personnel and vehicles were the largest cost drivers, followed by trainings and RDTs. The estimated cost per disability-adjusted life-years averted was $804, which in the context of Zambia was considered a highly cost-effective health intervention.

Data on feasibility and health system considerations were abstracted from two studies. Odero et al.,^28^ in a study linked to community-based MTaT in western Kenya,^17^^,^^18^ evaluated the feasibility of this mass campaign at the population level. Availability of health posts, community health visitors, available human resources at local health facilities, and health management information systems all made this intervention feasible. In a study linked to research conducted in Pakro subdistrict, Ghana,^23^ Ndong et al.^29^ assessed the perception of health workers and community members about the feasibility of MTaT. Overall, health workers and community participants perceived MTaT as a feasible intervention with many benefits, such as reducing incidence in children, increasing sensitization of the community about malaria, reducing hospital admissions, increasing work productivity, reducing expenditure for treatment, providing timely access to treatment at home, and reducing travel to health facilities.

### Mathematical modeling data.

Although mathematical modeling studies may have limited direct impact on generating empirical “data” for determining effect size and significance in recommendations, they have proven valuable in comprehending the dynamics of malaria transmission. These models, varying in complexity, contribute to decision-making in malaria control and elimination initiatives. Mathematical modeling helped in establishing operational criteria for MTaT, including aspects such as timing, frequency, coverage, and duration, which play a crucial role in informing strategies for achieving sustainable elimination levels. Furthermore, these studies provide modeling evidence for potential impacts of more sensitive diagnostic tests used in malaria screening. They have also contributed to identifying potential co-interventions that could maximize the benefits of MTaT.

A modeling study conducted by Mwesigwa et al.^30^ found that in low-transmission settings, three rounds of MTaT at the end of the dry season over 2 years, testing 85% of the population using highly sensitive RDTs, reduced malaria prevalence to a very low level. With additional concurrent or subsequent malaria control strategies, MTaT could lead to elimination. Rosas-Aguirre et al.^34^ modeled the intervention in low-transmission areas with PCD and symptomatic case management. The model predicted that three consecutive rounds of screening and treatment using polymerase chain reaction (PCR) at the beginning of the low-transmission season would result in the largest reduction, whereas repeated rounds of testing using PCR (over 5 years) or microscopy (over 10 years) would lower incidence and prevalence rates to near zero.

A modeling study by Gerardin et al.^33^ predicted that using MTaT in settings that had experienced recent reductions in the entomological inoculation rate (EIR) was more successful than implementation in areas that lacked insecticide-treated net (ITN) campaigns and had higher EIRs. Similar results were predicted by Kern et al.,^31^ who showed that MTaT sustained reductions over 3 years with a single intervention round in areas with lower endemicity (EIR <10 infectious bites per person per year [ib/p/y]). On the other hand, MTaT in areas with an EIR of more than 200 ib/p/y had the largest effect; however, the effects were not sustained if the intervention was not repeated in the subsequent year. Slater et al.^38^ evaluated the impact of different diagnostic thresholds on the likelihood of MTaT success. The simulation predicted that countries with an EIR below 4 ib/p/y could benefit from this intervention if the limit of detection of a diagnostic test was at least 2 parasites/μL. However, in countries where EIR is more than 4 ib/p/y, MTaT would not be an effective intervention even if diagnostic tests are highly sensitive.

A simulation by Stuckey et al.^36^ predicted that with currently available RDTs, low-density infections would persist after MTaT. Another modeling study by Gerardin et al.^35^ predicted that despite increasing the limit of detection of RDTs from 100 to 10 parasites/μL, low-density infections would remain undetected, thus leaving a substantial proportion of infections untreated.

A modeling study by Crowell et al.^32^ predicted that in terms of reducing transmission, MTaT was not cost-effective in low-transmission settings where the EIR was approximately 2 ib/p/y. However, in settings with an EIR of 20–50 ib/p/y and ITN coverage of 40–60%, the incremental cost-effectiveness ratio of MTaT was similar to that of scaling up ITN coverage. Thus, MTaT could be a suitable intervention in moderate- to high-transmission settings for reducing malaria burden, but it should be used as a complementary intervention to other recommended strategies. Millar et al.^8^ developed a spatial-explicit probabilistic model using publicly available data from six countries and estimated intervention costs considering different variables such as prevalence, diagnostic test performance, and sociodemographic data, including age and urbanicity. Millar et al.^8^ concluded that resource allocation could not be maximized with a “one-size-fits-all” approach because of substantial heterogeneity in malaria prevalence and performance of the diagnostic test.

## DISCUSSION

In areas seeking to reduce malaria transmission on the pathway to elimination, it may be necessary to implement strategies that target the identification and treatment of both symptomatic and asymptomatic infections. Active case detection strategies such as MTaT reach individuals who may be infected with the malaria parasite even if they are not experiencing symptoms and have not sought treatment. Because individuals are treated only if infected, ACD interventions are considered to be more acceptable to communities than chemoprevention strategies, even though they may be more difficult to implement. In addition, in contrast with chemoprevention (based on treatment administration regardless of infectious status), ACD strategies are thought to reduce unnecessary drug use. This, in turn, helps decrease drug costs and restricts the intervals of low drug concentrations in the bloodstream, likely lowering the risk of developing drug-resistant parasites. We conducted a systematic literature review and meta-analysis of MTaT to inform WHO guideline development about this strategy/its benefits and risks.

Overall, the evidence for outcomes in this review was based on a small number of studies. We excluded many studies because outcomes of interest were not reported. We reviewed four cRCTs conducted in Africa and Asia, and meta-analyses did not find MTaT associated with decreases in the incidence of infection, incidence of clinical malaria, or prevalence of malaria.

Community engagement prior to implementing MTaT campaigns was highlighted as one of the most important contextual factors, in line with the conclusions on MDA implementation experience.^40^ Furthermore, ensuring an adequate supply of commodities during such campaigns was essential to ensure the success and effectiveness of any such intervention. One of the main implementation challenges to deal with when targeting asymptomatic infections relates to social and cultural factors (i.e., the perception of subclinical malaria infections by local populations).^41^ However, contextual factors analysis revealed good acceptability of MTaT interventions among the study populations. In addition, mathematical modeling studies included in this review suggest that the effectiveness of MTaT depends on the transmission intensity, the diagnostic test threshold, the annual number and timing of MTaT rounds, and the total years for which the intervention was implemented. Moreover, other parallel interventions, including vector control tools, played an important role in the effectiveness of MTaT.

The intent of a “mass strategy” is to reduce human malaria transmission by implementing a timely and effective intervention in a population to a level where remaining cases are easily identifiable and can be eliminated with treatment.^9^ However, while implementing any effective strategy to reduce transmission to this low level, it is critical to consider asymptomatic cases or parasitemia. Mass testing and treatment is one such strategy that can reduce transmission to low levels if screening instruments are sensitive enough to detect low-density parasitemia.^42^ However, most of the trials assessed for this systematic review reported that the lack of a highly sensitive diagnostic tool in detecting low levels of parasitemia was their biggest challenge.

Studies that were excluded also could provide useful information for policymakers. One study^42^ conducted in Zanzibar in 2012 revealed that the success of an MTaT strategy depends on highly sensitive point-of-care diagnostic tools in pre-elimination settings. In another prospective study^43^ conducted in 2013–2014 in Central India, the prevalence of gametocytes in the tested population was higher in afebrile cases than in febrile cases, leading to the conclusion that asymptomatic cases can impede the elimination initiative.

The results of this systematic review were used by the WHO GDG and the Global Malaria Program to conditionally recommend against use of MTaT for reduction of malaria transmission.^9^ The limited number of studies contributing data on MTaT effectiveness assessed in this review have not shown significant evidence of an impact on malaria transmission. However, in some epidemiological contexts, MTaT implementation could still be a beneficial tool for reducing asymptomatic infections, measuring prevalence in the population, and providing overall improvements to malaria case management. The WHO did note, however, that MTaT might remain an appropriate strategy in very low–transmission settings where an MDA intervention is not considered acceptable. In addition, ACD remains an important surveillance strategy in areas or populations or at times when coverage of PCD is limited or nonexistent.

## Supplemental Materials

10.4269/ajtmh.23-0127 Supplemental Materials
