# Supplementary material for: Mass Testing and Treatment to Accelerate Malaria Elimination: A Systematic Review and Meta-Analysis
Source: Am J Trop Med Hyg. 2024 Mar 12;110(4 Suppl):44–53. doi: 10.4269/ajtmh.23-0127 (PMC10993795; doi:10.4269/ajtmh.23-0127)
Supplement: Supplemental Materials [file tpmd230127.SD1.pdf]

Supplemental Materials

Mass Testing and Treatment to Accelerate Malaria Elimination: A Systematic Review and Meta-Analysis

Beena Bhamani,[1*] Elisabet Martí Coma-Cros,[1] Maria Tusell,[1] Vita Mithi,[2-4] Elisa Serra,[1] Nana Williams,[1]

Kimberly A. Lindblade,[5] Koya C. Allen[1]

[1]Barcelona Institute for Global Health (ISGlobal), Hospital Clínic – University of Barcelona, Barcelona, Spain; [2] Armref Data for Action in Public Health Research Consultancy, Mzuzu, Malawi; [3]Society for Research on Nicotine and Tobacco-Genetics and Omics Network, Madison, USA; [4]Leaders of Africa Institute, Baltimore, USA; [5]Global Malaria Programme, World Health Organization, Geneva, Switzerland

[*]Address correspondence to Beena Bhamani, Barcelona Institute for Global Health (ISGlobal), Hospital Clínic - Universitat de Barcelona, Carrer Rosselló 171 Entl. 2a, Barcelona 08036. Email: beena.bhamani@isglobal.org

Supplementary Table 1. Search strategy

	**Search term**
1.	*malaria/ or Antimalarials/
2.	exp malaria, falciparum/ or exp malaria, vivax/
3.	malaria ovale.mp. or Plasmodium ovale/
4.	plasmodium malariae.mp. or Plasmodium malariae/
5.	1 or 2 or 3 or 4
6.	"mass test and treat" or Mass testing and treatment ". tw or MTAT.tw
7.	mass screening.tw or Mass screening/
8.	(screening or screened or diagnosed or diagnostics or test*).adj2 mass
9.	("case detection" or PACD or ACD).tw
10.	"Population-wide" or "Community-wide" adj2 (test* or treat* or screen*)
11.	"household screen*".tw
12.	"community case management".tw or CCM .tw
13.	"monthly screening and treatment".tw or MSAT.tw
14.	6 or 7 or 8 or 9 or 10 or 11 or 12 or 13
15.	5 and 1

Supplementary Table 2. List of studies excluded after full review and primary reasons for exclusion

Study	Reference	Reason for exclusion
Bal (2020)	1	Not retrieved
Balogh (2014)	2	Not retrieved - Abstract presented in 63rd Annual ASTMH meeting
Batwala (2011)	3	Intervention: febrile illness
Bennett (2019)	4	Not retrieved
Bousema (2013)	5	Crossed Referenced Article
Bousema (2016)	6	Excluded for Contextual Factors (Intervention: hotspot-targeted interventions with larviciding, distribution of long-lasting insecticide-treated nets, indoor residual spraying, and focal mass drug administration)
Briet (2017)	7	Not retrieved
Chaturvedi (2017)	8	Intervention: Mass screening to determine prevalence
Collins (2019)	9	Study protocol
Cisse (2013)	10	Study protocol – no publication found – contacted PI but no response
Conner (2020)	11	Intrevention: combination strategies i.e., MTaT with PECADOM++ (weekly fever screening, testing and treatment
Cook (2015)	12	Study focus area: testing RDTs vs PCR in MTaT
Crowell (2011)	13	Not retrieved
Crowell (2012)	14	Crossed Referenced Article-Primary article already included
Daniels (2020)	15	Outcomes: parasite relatedness and genomic diversity
Desai (2015)	16	Not retrieved
Dhiman (2011)	17	Intervention: fever screening/no assessment of impact of intervention
Diallo (2014)	18	Not retrieved
Diallo (2015)	19	Not retrieved
Drakeley (2018)	20	No output reported yet
Faye (2015)	21	Not retrieved
Griffin (2010)	22	Excluded for Mathematical Modelling (Intervention: MTaT using single dose of ACT)
Griffin (2012)	23	Not retrieved
Grueninger (2011)	24	Not retrieved
Guelbeogo (2011)	25	Not retrieved
Hamainza (2014)	26	Population: Monthly household visit by CHWs for screening in health facility

		catchment area, not whole population residing in delimited area/Excluded for Contextual Factors
Hamer (2020)	27	Crossed Referenced Article
Hennessee (2013)	28	Not retrieved
Kinzer (2010)	29	Study design: Only included those who tested negative in initial recruitment screening for follow-up in incidence study and treatment evaluation
Krishnamoorthy (1985)	30	Not retrieved
Landier (2018)	31	Intervention: testing febrile cases in communitty for early detection of cases
Larsen (2012)	32	Not retrieved
Larsen (2013)	33	Not retrieved/CRA
Liew (2018)	34	Study design: RCD, followed with active case detection through test and treat post outbreak; not a study
Lover (2018)	35	Not retrieved
Lover (2019)	36	Not retrieved
Macauley (2005)	37	Study focus area: Case studies review
Maude (2009)	38	Excluded for Contextual Factors (Study focus area: commentary)
Mlacha (2020)	39	Intervention: This is a reactive strategy conducting MTaT based on weekly data from HF and only targeting villages with higher incidences in previous week
Minja (2019)	40	Not retrieved
Mosha (2013)	41	Intervention: Screen and treat approach vs tMDA/MDA
Mwanga (2015)	42	Excluded for Mathematical Modelling (Intervention: combination strategies that does not include MTaT)
Mwesigwa (2019)	43	Not retrieved
Nankabirwa (2010)	44	Not retrieved
Nct (2016)	45	Not retrieved
Nikolov (2015)	46	Not retrieved
Nosten (2019)	47	Study protocol
Pactr (2013)	48	Not retrieved
Pang (2001)	49	Excluded for Contextual Factors Intervention: symptoms-based test and treat in target village)
Pradhan (2022)	50	Intervention-Comprehensive Case Management
Ndong (2017)	51	Crossed Referenced Article
Ndong (2020)	52	Study protocol
Samuels (2014)	53	Not retrieved

Samuels (2015)	[54]	Not retrieved/CRA
Samuels (2017)	[55]	Crossed Referenced Article
Samuels (2021)	[56]	Crossed Referenced Article
Scott (2015)	[57]	Not retrieved
Scott (2016)	[58]	Study design: Cross sectional study determining the risk factors for malaria and RDT positivity
Shirayama (2008)	[59]	Study design: survey
Silal (2014)	[60]	Excluded for Mathematical Modelling (Intervention: MDA and FTaT)
Silal (2018)	[61]	Not retrieved
Silumbe (2012)	[62]	Not retrieved
Slater (2018)	[63]	Not retrieved
Stresman (2020)	[64]	Intervention: Reactive strategies
Stuckey (2014)	[65]	Not retrieved
Stuckey (2016)	[66]	Excluded for Mathematical Modelling (CRA)
Sutanto (2015)	[67]	Not retrieved
Sutcliffe (2012)	[68]	Study design: Longitudinal cohort & Cross-sectional survey
Tairou (2015)	[69]	Not retrieved
Thanh (2015)	[70]	Intervention implementation criteria: Time frame does not include 1-24 months after the start of the intervention
Tiono (2013)	[71]	Crossed Referenced Article
Tiono (2014)	[72]	Study focus area: evaluation of diagnostics
Villegas (2010)	[73]	Not retrieved
Vitor-Silva (2016)	[74]	Study design: prospective cohort
Wenger (2013)	[75]	Not retrieved
Wenger (2014)	[76]	Not retrieved

1. Bal M, Das A, Ghosal J, Pradhan MM, Khuntia HK, Pati S, Dutta A, Ranjit M., 2020. Assessment of effectiveness of DAMaN: A malaria intervention program initiated by Government of Odisha, India. *PLoS One 15*

2. Balogh B, Hutton DW, Anupindi RM, Arney L, Liu M, Wu H, Larson PS, Yadav P, Wilson ML., 2014. Optimal coverage at minimal cost: A dynamic modeling approach to simultaneous allocation of multiple anti-malaria interventions. *63rd Annual Meeting of the American Society of Tropical Medicine and Hygiene, ASTMH*, 498

3. Batwala V, Magnussen P, Nuwaha F., 2011. Antibiotic use among patients with febrile illness in a low malaria endemicity setting in Uganda. *Malar J 10*

4. Bennett A, et al., 2019. Targeted surveillance for forest-based malaria transmission: results of a cluster randomized controlled trial in southern Lao PDR. *AJTMH 101*: 216

5. Bousema T, et al., 2013. The impact of hotspot-targeted interventions on malaria transmission: Study protocol for a cluster-randomized controlled trial. *Trials 14*

6. Bousema T, et al., 2016. The Impact of Hotspot-Targeted Interventions on Malaria Transmission in Rachuonyo South District in the Western Kenyan Highlands: A Cluster-Randomized Controlled Trial. *PLoS Med 13*

7. Briet OJT, Angluben R, Torno M, Navarro MAH, Deray R, Schapira A., 2017. Modelling to support the planning of malaria elimination in southern Palawan, the Philippines. *Tropical Medicine and International Health*, 55

8. Chaturvedi N, Krishna S, Bharti PK, Gaur D, Chauhan VS, Singh N., 2017. Prevalence of afebrile parasitaemia due to Plasmodium falciparum & P. vivax in district Balaghat (Madhya Pradesh): Implication for malaria control. *Indian J Med Res 146*: 260

9. Collins KA, Ouedraogo A, Guelbeogo WM, Awandu SS, Stone W, Soulama I, Ouattara MS, Nombre A, Diarra A, Bradley J, Selvaraj P, Gerardin J, Drakeley C, Bousema T, Tiono A., 2019. Investigating the impact of enhanced community case management and monthly screening and treatment on the transmissibility of malaria infections in Burkina Faso: study protocol for a cluster-randomised trial. *BMJ Open 9*: e030598

10. Cisse B, et al., 2013. Randomized trial of spatially targeted malaria control to virtually eliminate malaria in areas of very low incidence and patchy transmission in Senegal. Available at: https://trialsearch.who.int/?trialid=PACTR201310000575267. Accessed. 2013

11. Conner RO, et al., 2020. Mass testing and treatment for malaria followed by weekly fever screening, testing and treatment in Northern Senegal: Feasibility, cost and impact. *Malar J 19*

12. Cook J, Xu W, Msellem M, Vonk M, Bergström B, Gosling R, Al-Mafazy AW, McElroy P, Molteni F, Abass AK, Garimo I, Ramsan M, Ali A, Mårtensson A, Björkman A., 2015. Mass screening and treatment on the basis of results of a plasmodium falciparum-specific rapid diagnostic test did not reduce malaria incidence in zanzibar. *Journal of Infectious Diseases 211*: 1476–1483

13. Crowell V, Hardy D, Chitnis N, Maire N, Smith T., 2011. Mass screening and treatment for P. falciparum malaria: How effective is it likely to be? *Tropical Medicine and International Health*

14. Crowell V, Briëf OJ, Hardy D, Chitnis N, Maire N, di Pasguale A, Smith TA., 2012. Modeling the cost-effectiveness of mass screening and treatment for reducing Plasmodium falciparum malaria burden. *Malaria Journal 2012 11:1 11*: 1–1

15. Daniels RF, et al., 2020. Evidence for reduced malaria parasite population after application of population-level antimalarial drug strategies in Southern Province, Zambia. *American Journal of Tropical Medicine and Hygiene 103*: 66–73

16. Desai M, et al., 2015. Impact of intermittent mass screening and treatment (IMSAT) for malaria on incidence of infection in an area of high malaria transmission in Western Kenya. *AJTMH 93*: 180

17. Dhiman S, Gopalakrishnan R, Goswami D, Rabha B, Baruah I, Singh L., 2011. Malaria incidence among paramilitary personnel in an endemic area of Tripura. *Indian J Med Res 133*: 665

18. Diallo A, et al., 2014. A cluster-randomized trial of targeted control to eliminate malaria in central Senegal: study design and acceptability of the interventions. *63rd Annual Meeting of the American Society of Tropical Medicine and Hygiene, ASTMH*, 198

19. Diallo A, et al., 2015. A cluster-randomized trial of targeted control to eliminate malaria in central Senegal: main results in year 2. *64th Annual Meeting of the American Society of Tropical Medicine and Hygiene, ASTMH*, 81

20. Anon. P. Falciparum Infection Dynamics and Transmission to Inform Elimination (INDIE-1a) - Full Text View - ClinicalTrials.gov. Available at: https://www.clinicaltrials.gov/ct2/show/NCT03705624?term=NCT03705624&draw=2&rank=1. Accessed

21. Faye F, et al., 2015. Mass testing and treatment for malaria followed by weekly visits to screen for fever cases in low transmission areas in matam and louga regions, Senegal: A pilot study. *64th Annual Meeting of the American Society of Tropical Medicine and Hygiene, ASTMH*, 497

22. Griffin JT, et al., 2010. Reducing Plasmodium falciparum malaria transmission in Africa: A model-based evaluation of intervention strategies. *PLoS Med 7*

23. Griffin JT, et al., 2012. Strategies towards plasmodium falciparum malaria elimination in Africa using currently available tools. *61st Annual Meeting of the American Society of Tropical Medicine and Hygiene, ASTMH*, 262

24. Grueninger H, Rietveld H., 2011. Ten years experience with Coartem: A patient-centric approach to fighting malaria. *60th Annual Meeting of the American Society of Tropical Medicine and Hygiene, ASTMH*, 114

25. Guelbeogo WM, N'Fale S, Lucas J., 2011. Impact of treating Plasmodium falciparum asymptomatic carriers on the dynamic of malaria transmission. *7th European Congress on Tropical Medicine and International Health*, 147

26. Hamainza B, Moonga H, Sikaala CH, Kamuliwo M, Bennett A, Eisele TP, Miller J, Seyoum A, Killeen GF., 2014. Monitoring, characterization and control of chronic, symptomatic malaria infections in rural Zambia through monthly household visits by paid community health workers. *Malar J 13*

27. Hamer DH, Miller JM., 2021. Why Did Mass Test and Treat Have No Effect on Malaria Prevalence in Western Kenya? *Clinical Infectious Diseases 72*: 1936–1937

28. Hennessee IP, Linn A, Ndiaye Y, Diop IL, Tandian CM., 2013. Pecadom plus: Increasing care access and decreasing morbidity in rural southeast senegal through active, home-based surveillance and treatment of malaria. *62nd Annual Meeting of the American Society of Tropical Medicine and Hygiene, ASTMH*, 65

29. Kinzer MH, Chand K, Basri H, Lederman ER, Susanti AI, Elyazar I, Taleo G, Rogers WO, Bangs MJ, Maguire JD., 2010. Active case detection, treatment of falciparum malaria with combined chloroquine and sulphadoxine/pyrimethamine and vivax malaria with chloroquine and molecular markers of anti-malarial resistance in the Republic of Vanuatu. *Malar J 9*

30. Krishnamoorthy K, Jambulingam P, Sabesan S, Rajendran G, Gunasekaran K., 1985. Mass blood survey in three villages of Rameswaram Island endemic for malaria. *Indian J Med Res 81*: 140–142

31. Landier J, et al., 2018. Effect of generalised access to early diagnosis and treatment and targeted mass drug administration on Plasmodium falciparum malaria in Eastern Myanmar: an observational study of a regional elimination programme. *The Lancet 391*: 1916–1926

32. Larsen DA, Miller JM, Keating J, Yukich J, Hamainza B, Moonga H, Silumbe K, Lungu C, Chirwa J, Eisele TP., 2012. The effectiveness of a single round of mass malaria screening and treatment in southern Zambia. *61st Annual Meeting of the American Society of Tropical Medicine and Hygiene, ASTMH*, 140

33. Larsen DA, Miller JM, Keating J, Yukich J, Shaffer J, Hamainza B, Silumbe K, Eisele T., 2013. Village-level characteristics associated with spatial distributions of malaria-infected individuals in an area of Southern Zambia receiving mass screening and treatment. *62nd Annual Meeting of the American Society of Tropical Medicine and Hygiene, ASTMH*, 435

34. Liew JWK, et al., 2018. Importance of Proactive Malaria Case Surveillance and Management in Malaysia. *Am J Trop Med Hyg 98*: 1709

35. Lover AA, et al., 2018. A community-randomized trial assessing the effectiveness of targeted active malaria case detection among high-risk populations in southern Lao PDR: study design and baseline survey results. *67th Annual Meeting of the American Society of Tropical Medicine and Hygiene, ASTMH*, 140

36. Lover AA, et al., 2019. Study protocol for a cluster-randomized split-plot design trial to assess the effectiveness of targeted active malaria case detection among high-risk populations in Southern Lao PDR (the AcME-Lao study). *Gates Open Res 3*: 1730

37. Macauley C., 2005. Aggressive active case detection: A malaria control strategy based on the Brazilian model. *Soc Sci Med 60*: 563–573

38. Maude RJ, Pontavornpinyo W, Saralamba S, Dondorp AM, Day NPJ, White NJ, White LJ., 2009. The role of mathematical modelling in malaria elimination and eradication (Comment on: Can malaria be eliminated?). *Trans R Soc Trop Med Hyg 103*: 643–644

39. Mlacha YP, et al., 2020. Effectiveness of the innovative 1,7-malaria reactive community-based testing and response (1, 7-mRCTR) approach on malaria burden reduction in Southeastern Tanzania. *Malar J 19*: 1–12

40. Minja EG, Swai JK, Mrimi E, Ngowo H, Okumu F., 2019. Prevalence and drivers of plasmodium infection across villages in Southeastern Tanzania. *68th Annual Meeting of the American Society of Tropical Medicine and Hygiene, ASTMH*, 94

41. Mosha JF, et al., 2013. Epidemiology of subpatent Plasmodium falciparum infection: Implications for detection of hotspots with imperfect diagnostics. *Malar J 12*

42. Mwanga GG, Haario H, Capasso V., 2015. Optimal control problems of epidemic systems with parameter uncertainties: Application to a malaria two-age-classes transmission model with asymptomatic carriers. *Math Biosci 261*: 1–12

43. Mwesigwa J, et al., 2019. Field performance of the highly-sensitivity rapid diagnostic test in a setting of highly seasonal malaria transmission. *The Royal Society of Tropical Medicine and Hygiene 113*: S13

44. Nankabirwa JI, Banek K, DiLiberto D, Taaka L, Chandler C, Staedke S., 2010. Community-based delivery of health care: What is the capacity for expanding interventions? *59th Annual Meeting of the American Society of Tropical Medicine and Hygiene, ASTMH*, 391

45. Desai MR, et al., 2016. Evaluation of Community-based Mass Screening and Treatment for Malaria in Western Kenya. Available at: https://clinicaltrials.gov/ct2/show/NCT02987270. Accessed. 2016

46. Nikolov M, Gerardin J, Bever CA, Eckhoff PA, Wenger EA., 2015. Modeling the effectiveness of population-level malaria infection detection strategies for optimal campaign scoping. *64th Annual Meeting of the American Society of Tropical Medicine and Hygiene, ASTMH*, 293

47. Nosten F, et al., 2019. Mass Screening and Treatment for Reduction of Falciparum Malaria. Available at: https://clinicaltrials.gov/ct2/show/NCT04093765?term=NCT04093765&draw=2&rank=1. Accessed. 2019

48. Cisse B, et al., 2013. A trial of targetted control to eliminate malaria in Central Senegal. Available at: https://www.cochranelibrary.com/central/doi/10.1002/central/CN-01863710/full. Accessed. 2013

49. Pang LW, Piovesan-Alves F., 2001. Economic advantage of a community-based malaria management program in the Brazilian Amazon. *Am J Trop Med Hyg 65*: 883–886

50. Pradhan MM, et al., 2022. Impact of the malaria comprehensive case management programme in Odisha, India. *PLoS One 17*: e0265352

51. Ndong IC, et al., 2019. Impact of Scaling up Mass Testing, Treatment and Tracking on Malaria Prevalence Among Children. Available at: https://clinicaltrials.gov/ct2/show/NCT04167566. Accessed. 2019

52. Ndong IC, et al., 2020. Determining the Impact of Scaling up Mass Testing, Treatment and Tracking on Malaria Prevalence in Ghana. Available at: https://clinicaltrials.gov/ct2/show/NCT04301531?term=NCT04301531&draw=2&rank=1. Accessed. 2020

53. Samuels AM, Odero NA, Onyango W, Otieno K, Otieno P, Shi YP, Hamel MJ, Lindblade K, Kariuki S, Desai M., 2014. Baseline epidemiological characteristics of participants enrolled in a trial of intermittent mass screening and treatment for malaria in Western Kenya. *63rd Annual Meeting of the American Society of Tropical Medicine and Hygiene, ASTMH*, 458

54. Samuels AM, et al., 2015. Impact of intermittent mass screening and treatment (iMSaT) on community malaria parasitemia prevalence in an area of high transmission-Kenya 2013-2014. *64th Annual Meeting of the American Society of Tropical Medicine and Hygiene, ASTMH*, 278

55. Samuels AM, et al., 2017. Community-based intermittent mass testing and treatment for malaria in an area of high transmission intensity, western Kenya: study design and methodology for a cluster randomized controlled trial. *Malar J 16*: 1–12

56. Samuels AM, et al., 2021. Mass testing and treatment on malaria in an area of western Kenya. *Clinical Infectious Diseases 72*: 1103–1104

57. Scott C, et al., 2015. Mass testing and treatment for malaria in moderate transmission areas in Amhara region, Ethiopia. *64th Annual Meeting of the American Society of Tropical Medicine and Hygiene, ASTMH 93*: 471

58. Scott CA, et al., 2016. Mass testing and treatment for malaria in low transmission areas in Amhara Region, Ethiopia. *Malar J 15*

59. Shirayama Y, Phompida S, Kuroiwa C., 2008. Monitoring malaria control in Khammouane province, Laos: an active case detection survey of Plasmodium falciparum malaria using the Paracheck rapid diagnostic test. *Trans R Soc Trop Med Hyg 102*: 743–750

60. Silal SP, Little F, Barnes KI, White LJ., 2014. Towards malaria elimination in Mpumalanga, South Africa: A population-level mathematical modelling approach. *Malar J 13*: 1–12

61. Silal SP, White LJ, Kollipara A, Moya M, Graffy R, Mabunda E, Malatje G, Qwabe B, Pillay Y, Moonasar D., 2018. Supporting decision-making for malaria elimination in south Africa: A mathematical modelling approach. *67th Annual Meeting of the American Society of Tropical Medicine and Hygiene, ASTMH*, 339

62. Silumbe K, Larsen D, Hamainza B, Miller JM, Lungu C, Hawela M, Chirwa J, Kamuliwo M., 2012. Steps towards malaria elimination: Integrating population-wide test and treat campaigns into malaria control in zambia. *61st Annual Meeting of the American Society of Tropical Medicine and Hygiene, ASTMH*, 357–358

63. Slater HC, Li R, Walker PG, Ghani A., 2018. Modelling the impact of an ultra-sensitive plasmodium falciparum rapid diagnostic test (U-RDT): Detecting asymptomatic infections and the potential for overtreatment. *67th Annual Meeting of the American Society of Tropical Medicine and Hygiene, ASTMH*, 540–541

64. Stresman G, Whittaker C, Slater HC, Bousema T, Cook J., 2020. Quantifying Plasmodium falciparum infections clustering within households to inform household-based intervention strategies for malaria control programs: An observational study and meta-analysis from 41 malaria-endemic countries. *PLoS Med 17*

65. Stuckey EM, Miller JM, Littrell M, Chitnis N, Steketee RW., 2014. Investigating operational strategies for antimalarial drug administration in Zambia's Southern province: A simulation study. *63rd Annual Meeting of the American Society of Tropical Medicine and Hygiene, ASMTH*, 461

66. Stuckey EM, Miller JM, Littrell M, Chitnis N, Steketee R., 2016. Operational strategies of anti-malarial drug campaigns for malaria elimination in Zambia's southern province: A simulation study. *Malar J 15*

67. Sutanto I, et al., 2015. Mass screening and treatment does not impact malaria incidence in West Timor, Indonesia. *64th Annual Meeting of the American Society of Tropical Medicine and Hygiene, ASTMH*, 263

68. Sutcliffe CG, Kobayashi T, Hamapumbu H, Shields T, Mharakurwa S, Thuma PE, Louis TA, Glass G, Moss WJ., 2012. Reduced risk of malaria parasitemia following household screening and treatment: A cross-sectional and longitudinal cohort study. *PLoS One 7*

69. Tairou F, Circe Ba E, Diallo A, Sy O, Cisse B, Gomis J, Gaye O, Milligan P, Pitt C., 2015. The costs and cost-effectiveness of two spatially targeted, multi-component malaria elimination strategies: results of a large three-arm cluster-randomized trial in rural Senegal. *64th Annual Meeting of the American Society of Tropical Medicine and Hygiene, ASMTH*, 81

70. Thanh PV, et al., 2015. Epidemiology of forest malaria in Central vietnam: The hidden parasite reservoir. *Malar J 14*

71. Tiono AB, Guelbeogo MW, Sagnon NF, Nébié I, Sirima SB, Mukhopadhyay A, Hamed K., 2013. Dynamics of malaria transmission and susceptibility to clinical malaria episodes following treatment of Plasmodium falciparum asymptomatic carriers: results of a cluster-randomized study of community-wide screening and treatment, and a parallel entomology study. *BMC Infect Dis 13*: 535

72. Tiono AB, Ouédraogo A, Diarra A, Coulibaly S, Soulama I, Konaté AT, Barry A, Mukhopadhyay A, Sirima SB, Hamed K., 2014. Lessons learned from the use of HRP-2 based rapid diagnostic test in community-wide screening and treatment of asymptomatic carriers of Plasmodium falciparum in Burkina Faso. *Malar J 13*

73. Villegas L, et al., 2010. Mass screening and treatment for malaria among gold miners in Suriname. *International Journal of Infectious Diseases 14*: e435

74. Vitor-Silva S, et al., 2016. Declining malaria transmission in rural Amazon: Changing epidemiology and challenges to achieve elimination. *Malar J 15*

75. Wenger EA, Eckhoff PA, Littrell M, Silumbe K, Hamainza B, Miller JM, Steketee RW., 2013. Modeling for malaria control and elimination scenario planning: Application of the epidemiological modeling (EMOD) malaria disease transmission kernel to community-based intervention delivery in Southern Zambia. *62nd Annual Meeting of the American Society of Tropical Medicine and Hygiene, ASTMH*, 396

76. Wenger EA, Upfill-Brown AM, Gerardin JL, Eckhoff PA., 2014. Spatial dynamics of malaria transmission in the EMOD model for campaigns targeting sustained regional elimination in Southern Zambia. *63rd Annual Meeting of the American Society of Tropical Medicine and Hygiene, ASTMH*, 14

Supplementary Figure 1. Risk of Bias summary for the outcomes assessed in the cRCTs

Study ID	Outcome	D1a	D1b	D2	D3	D4	D5	Overall
Larsen 2015	Incidence of clinical malaria community	+	+	+	+	+	+	+
Larsen 2015	Prevalence of infection community	+	+	+	+	+	+	+
Samuels 2020	Prevalence of infection community	+	+	+	+	+	+	+
Desai 2020	Incidence of infection community	+	+	+	+	+	+	+
Desai 2020	Inicidence of clinical malaria community	+	+	+	+	+	+	+
Sutanto 2018	Incidence of infection community	+	+	+	+	+	+	+
Sutanto 2018	Adverse events	+	+	+	+	+	!	!
Tiono 2013	Prevalence of infection community	+	+	+	+	+	+	+
Tiono 2013	AEs including SAEs	+	+	+	+	+	!	!
Tiono 2013	Incidence of clinical malaria community	+	+	+	+	+	+	+

\+ Low risk
! Some concerns
\- High risk

Supplementary Figure 2: Risk of Bias summary for the cRCTs by percentage

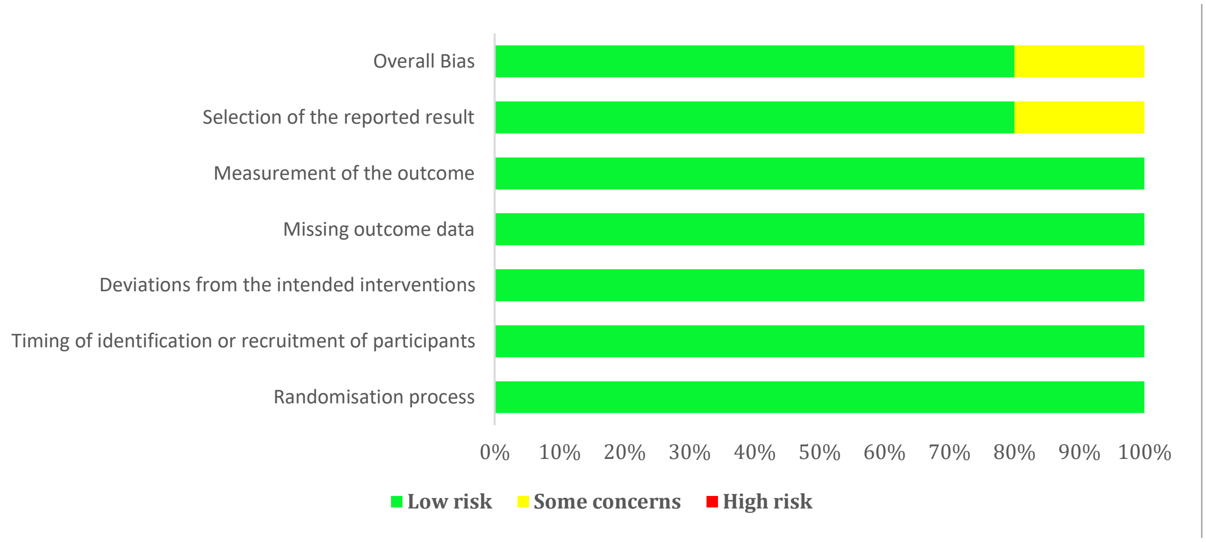

Supplementary Figure 3: Risk of Bias summary for the controlled before and after study

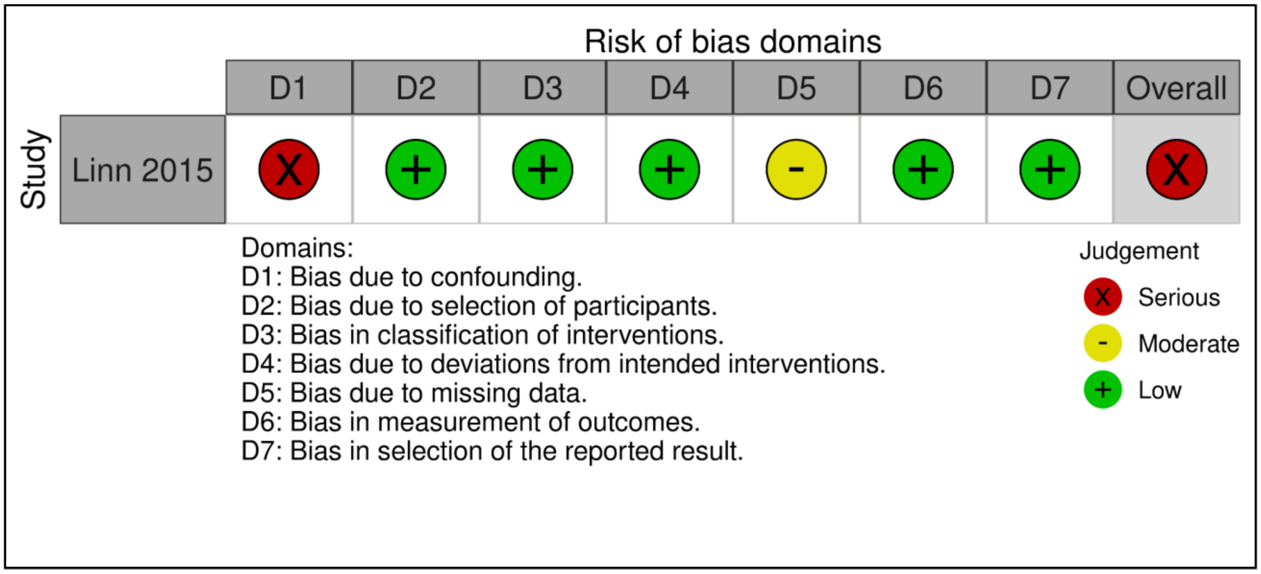

Supplementary Table 3. Data extracted from included studies

Study 1 Desai et al. and Samuels et al. (2020)

**Study characteristics**	
METHODS	
Study dates	2013-2015
Location	Siaya district, western Kenya
Peak transmission season	May to July<br>November to December
Baseline transmission intensity	High, baseline incidence rate 0.20 cases/p-y in intervention and 0.21 cases/p-y in control<br>cluster parasite prevalence (median) was 33.9% in the intervention group and 36.8% in the control group
Parasite species	Plasmodium falciparum
Vector species	Anopheles arabiensis, Anopheles funestus,
Study design	Cluster-randomized controlled trial
Statistical power calculation	Incidence- Assuming a baseline incidence of 1.6 infections per person per year, a type I error rate of 5%, and 80% power to detect a difference in malaria incidence between intervention and control arms of at least 30%. The study required 220 per arm each year and author adjusted the sample size to 330 per arm for an expected 18% loss.<br>Prevalence- assuming a malaria infection prevalence of 40% in the control arm, a type I error rate of 5%, and 80% power to detect a relative difference in malaria prevalence of 50% between arms, 20 compounds per cluster were selected to attain calculated sample size
Clusters	Unit of randomization:<br>MTaT-wedges consisting of villages<br>Incidence- Cohort of residents from 400 compunds<br>Prevalence- 20 compounds from 20 clusters<br>Features of the clusters: Buffer areas, restricted randomization<br>Number of clusters selected: 30<br>Number of clusters analyzed: 20<br>Average cluster size: 10 villages
PARTICIPANTS	
Targeted population	Total: all residents of 10 HF catchment area<br>Intervention:<br>Incidence- Longitudinal cohort study: 330 participants<br>Prevalence-Cross-sectional surveys: 20 compounds from the core area per cluster (857 participants)<br>Comparison:<br>Incidence: Longitudinal cohort study: 330 participants<br>Prevalence: Cross-sectional surveys: 20 compounds from the core area per cluster (857 participants)
Participant characteristics	MTaT: all inhabitants >1 month<br>Longitudinal cohort studies: ≥1 year old<br>Cross-sectional studies: ≥1 month old

INTERVENTION	
Intervention	Mass testing and treatment
Comparator	Standard of care
Background interventions	Long lasting insecticide nets (in both arms)
Drug and manufacturer	Dihydroartemisinin–piperaquine (DP; Duocotexcin, Holy-Cotec, China, or Eurartesim, Sigma-Tau, Italy); children aged 1-3 months treated with artemether–lumefantrine (AL): pregnant women with quinine
Dosage	40 mg dihydroartemisinin and 320 mg piperaquine for 6 years and above; 20mg dihydroartemisinin and 160mg for children > 3 months to 5 years
Number of rounds per season or year	3
Treatment interval	Every 4 months
Duration of the intervention	2 months
Treatment adherence	Round 1: 350/371 (94.34%), Round 2: 323/341 (94.72%), Round 3: 291/318 (91.51%), Round 4: 331/347 (95.39%), Round 5: 363/382 (95.03%), Round 6: 303/325 (93.23%)
OUTCOMES	
Incidence of malaria infection at the community level	Measurement: Longitudinal cohort, measured by microscopy/RDT<br>Timepoints: 0-12 months<br>Sample size:<br>year 1: 516 (MTaT 253 & Control 263)<br>year 2: 550 (MTaT 272 & Control 278)
Incidence of clinical malaria at the community level	Measurement: Passive surveillance, measured by RDT<br>Timepoints: 0-12 months<br>Sample size: N/A
Prevalence of infection at the community level	Measurement: Cross-sectional survey<br>Timepoints: 2 months post intervention (third round of MTaT)<br>Sample size:<br>year 1: MTaT 179 (896 individuals) compounds & control 183 compounds (1016 individuals)<br>year 2: MTaT 179 (841 individuals) compounds & control 190 compounds (907 individuals)

Study 2 Larsen et al. (2015)

**Study characteristics**	
METHODS	
Study dates	Dec 2011-July 2013
Location	Gwembe, southern Kalomo, Siavonga, and Sinazongwe districts in southern Zambia
Peak transmission season	March-May
Baseline transmission intensity	Moderate, malaria parasite prevalence in children 1–59 months of age was 34.5% in the MTAT and 38.5% in control

	monthly confirmed case incidence in the preintervention period was 33.4 per 1,000 catchment population in control areas and 36.2 per 1,000 catchment population in intervention areas
Parasite species	Plasmodium falciparum
Vector species	Anopheles arabiensis and Anopheles funestus
Study design	Cluster randomized step-wedge control trial
Statistical power calculation	total sample size of 3,000 children < 5 years of age in 2,100 households > 2 rounds were sought to effectively measure a 50% reduction in malaria parasite prevalence between intervention and control groups, from an assumed 10% malaria parasite prevalence at baseline with a design effect of two.
Clusters	Unit of randomization: 46 HFCA, organized into 18 contiguous randomization group<br>Features of the clusters: Satellite imagery from google earth<br>Number of clusters selected: 18<br>Number of clusters analyzed: 18<br>Average cluster size: 2-3 HFCA
PARTICIPANTS	
Targeted population	MTaT & Incidence: All inhabitants of selected districts<br>Prevalence: 3000 in 2100 households
Participant characteristics	Prevalence: children 1-59 months
INTERVENTION	
Intervention	Mass testing and treatment
Comparator	Standard of care
Background interventions	ITNs and IRS
Drug and manufacturer	Artemether-lumefantrine (AL) as per recommendation by MOH
Dosage	As per national guidelines provided by MOH
Number of rounds per season or year	3
Treatment interval	Every other month
Duration of the intervention	1 year
Treatment adherence	No monitoring was done for treatment adherence
OUTCOMES	
Incidence of clinical malaria at the community level	Measurement: HMIS based outpatient malaria case incidence<br>Timepoints: 0-12 months<br>Sample Size: All inhabitants of HFCA
Prevalence of infection at the community level	Measurement: Malaria indicator household survey, by RDT<br>Timepoints: 6 months post-intervention<br>Sample size: 513 (intervention); 511 (control)

Study 3 Sutanto et al. (2018)

**Study characteristics**	
METHODS	
Study dates	2013
Location	Wesiku district, West Timor, Indonesia
Peak transmission season	April-November
Baseline transmission intensity	Low, SPR in MST3 vs MST2 was 7.4% vs 8.7%
Parasite species	Plasmodium falciparum, Plasmodium vivax & Plasmodium malariae
Vector species	Anopheles barbirostris, Anopheles subpictus and Anopheles vagus
Study design	open-label, community-wide cluster-randomized controlled trial
Statistical power calculation	The recruitment target was 1029 subjects per arm, considering a cluster design effect of 1.5, 5% significance, 80% power, and a 1:1 sample size ratio between intervention and control arms. A target of 115 children per arm would yield a power of 82% in detecting an estimated 50% reduction in malaria incidence following MST.
Clusters	Unit of randomization: Households<br>Features of the clusters: Stratified<br>Number of clusters selected: 16<br>Number of clusters analyzed: 11 for prevalence & 16 for incidence<br>Average cluster size:<br>Average Households in clusters MST3-28, MST2-20, MST0-37<br>Average Resident in clusters MST3-89, MST2-71, MST0-124
PARTICIPANTS	
Targeted population	Total: All inhabitants for selected disctrict<br>Intervention: Prevalence 1029, Incidence 115 children<br>Comparison: Prevalence 1029, Incidence 115 children
Participant characteristics	Prevalence: all inhabitants<br>Incidence: school children
INTERVENTION	
Intervention	Mass Testing and Treatment (MST)
Comparator	Standard of care
Background interventions	None
Drug and manufacturer	DHP (fixed-dose tablets of 40 mg dihydroartemisinin, 320 mg piperaquine; D-ARTEPP, Guilin Pharmaceutical Co, China, 6 December 2014 expiry); primaquine (15-mg primaquine base tablets; PT Phapros Tbk, Jakarta, Indonesia, October 2014 expiry)
Dosage	DHP regimen was daily based on patients' weight. For P. falciparum, primaquine was given as a single dose on day 1 as per patients' weight. For P. vivax infection the primaquine dose daily for 14 days as per patient weight

Number of rounds per season or year	MST3- 3 rounds<br>MST2- 2 rounds
Treatment interval	MST3-5 weeks<br>MST2-10 weeks
Duration of the intervention	6 months
Treatment adherence	Drug adherence was defined as taken completely as prescribed with witnessing, and occurred with >90% of cases
OUTCOMES	
Incidence of malaria infection at the community level	Measurement: Microscopy and PCR<br>Timepoints: 0-6 months<br>Sample size: 124 (MST3); 57 (MST2); 143 (MST0)
AEs	Drug administration during this study did not prompt withdrawal of any subject, and no serious AEs occurred. The most common AEs during treatment were fever (0.023/person-day), headache (0.008/person-day), vomiting (0.006/person-day), cough (0.004/person-day), shivering (0.003/person-day), and nasal congestion (0.002/person-day).

Study 4 Tiono et al. (2013)

**Study characteristics**	
METHODS	
Study dates	January 2011 to January 2012
Location	Saponé district, Burkina Faso
Peak transmission season	June-November
Baseline transmission intensity	High, mean prevalence of asymptomatic carriers intervention 42.8% & control 47.5%
Parasite species	Plasmodium falciparum
Vector species	Not mentioned
Study design	single-centre, controlled, parallel, cluster-randomized
Statistical power calculation	Not provided
Clusters	Unit of randomization: Villages<br>Features of the clusters: Stratified randomization<br>Number of clusters selected: 118<br>Number of clusters analyzed: 18<br>Average cluster size: 1 village
PARTICIPANTS	
Targeted population	Total: All inhabitants for selected district<br>Intervention: 6817<br>Comparison: 7258
Participant characteristics	Prevalence: all inhabitants<br>Incidence: children <5 years
INTERVENTION	

Intervention	Community-wide screening and treatment
Comparator	Standard of care
Background interventions	LLINs
Drug and manufacturer	AL/AL dispersible (20 mg artemether and 120 mg lumefantrine)
Dosage	twice a day for three consecutive days as per body weight
Number of rounds per season or year	3
Treatment interval	Every month
Duration of the intervention	1 year
Treatment adherence	Not quantified- AL adherence was good in those identified as asymptomatic carriers and in individuals with symptomatic malaria.
OUTCOMES	
Incidence of clinical malaria at the community level	Measurement: Microscopy/RDT<br>Timepoints: 0-12 months<br>Sample size: intervention: 5897, control: 6510
AEs including SAEs	There were no notable differences in AEs or SAEs between the intervention arm and the control arm at either the cluster or individual level and no new or unexpected safety findings were recorded. In total, 0.3% of treated asymptomatic carriers reported at least one AE within 7 days of starting treatment.
Prevalence of infection at the community level	Measurement: Microscopy<br>Timepoints: 9 months post-intervention<br>Sample size: intervention: 5897, control: 6510

Study 5 Linn et al. (2015)

**Study characteristics**	
METHODS	
Study dates	July to November 2013
Location	Saraya health district, South-east Senegal
Peak transmission season	July-November
Baseline transmission intensity	High, mean parasite prevalence of symptomatic malaria-intervention 1.88% and control 1.58%
Parasite species	Plasmodium falciparum
Vector species	Not mentioned
Study design	Quasi-experimental study design
Statistical power calculation	Not applicable
Clusters	N/A
PARTICIPANTS	

Targeted population	Total: 8954<br>Intervention: 4217<br>Comparison: 8954
Participant characteristics	All individuals of intervention households with symptoms of malaria
INTERVENTION	
Intervention	ProACT
Comparator	Passive community case management
Background interventions	LLINs & SMC
Drug and manufacturer	ACT
Dosage	As per national malaria control programme (NMCP)
Number of rounds per season or year	21
Treatment interval	Weekly
Duration of the intervention	21 weeks
Treatment adherence	Not mentioned
OUTCOMES	
Prevalence of infection at the community level	Measurement: symptomatic screening with RDT<br>Timepoints: 21 weeks<br>Sample size: 3762

Study 6 Ndong et al. (2019)

**Study characteristics**	
METHODS	
Study dates	2017-2018
Location	Pakro sub-district, Ghana
Peak transmission season	Perennial
Baseline transmission intensity	In July 2017, the prevalence of asymptomatic parasitemia was 36.3%
Parasite species	Plasmodium falciparum
Vector species	Not mentioned
Study design	Uncontrolled before and after
Statistical power calculation	Not applicable
Clusters	Not applicable
PARTICIPANTS	
Targeted population	Total: 5000

Participant characteristics	All inhabitants of selected area
INTERVENTION	
Intervention	Mass testing, treating and tracking (MTTT)
Comparator	Not applicable
Background interventions	Community case management
Drug and manufacturer	ACT
Dosage	As per the guidelines of National malaria control programme (NMCP)
Number of rounds per season or year	4
Treatment interval	4 months
Duration of the intervention	1 year
Treatment adherence	No data provided
OUTCOMES	
Prevalence of infection (asymptomatic parasitemia)	Measurement: RDT<br>Timepoints: Every four months at each round<br>Sample size: Round 1: 3891 & Round 4: 4941
Prevalence of infection (symptomatic parasitemia)	Measurement: RDT<br>Timepoints: 1 year

Study 7 Bharti et al. (2020)

**Study characteristics**	
METHODS	
Study dates	2017 to 20020
Location	Mandla, Madhya Pradesh, India
Peak transmission season	June to September
Baseline transmission intensity	Varied with different annual parasitic incidence (API); Malaria prevalence 123 per 100,000
Parasite species	Plasmodium falciparum
Vector species	Not mentioned
Study design	Uncontrolled before and after
Statistical power calculation	Not applicable
Clusters	Not applicable
PARTICIPANTS	
Targeted population	Total: 63,194

Participant characteristics	All inhabitants of selected area
INTERVENTION	
Intervention	Mass Screening and Treatment (MSAT)
Comparator	Not applicable
Background interventions	ITN, IRS, active case management
Drug and manufacturer	ACT
Dosage	As per the national drugs policy
Number of rounds per season or year	3
Treatment interval	-
Duration of the intervention	-
Treatment adherence	-
OUTCOMES	
Prevalence of infection	Measurement: RDT<br>Three rounds of MTaT were conducted to determine prevalence in the asymptomatic reservoir. MTaT was compared with detection through passive surveillance prevalence. 1st round-moderate to high burden areas - 50/28,527 i.e. 0.18% vs 0.06% from passive surveillance; 2nd round-low to high burden areas - 7/11,363 i.e. 0.06% vs 0.03% from passive surveillance; 3rd round- RCD of cryptic cases in 50 households -3/8,467 i.e. 0.03%
